# Fluorescence Quenching Studies on the Interactions between Chosen Fluoroquinolones and Selected Stable TEMPO and PROXYL Nitroxides

**DOI:** 10.3390/ijms22020885

**Published:** 2021-01-17

**Authors:** Krzysztof Żamojć, Irena Bylińska, Wiesław Wiczk, Lech Chmurzyński

**Affiliations:** Faculty of Chemistry, University of Gdańsk, Wita Stwosza 63, 80-308 Gdańsk, Poland; irena.bylinska@ug.edu.pl (I.B.); wieslaw.wiczk@ug.edu.pl (W.W.); lech.chmurzynski@ug.edu.pl (L.C.)

**Keywords:** fluoroquinolone antibiotics, stable TEMPO and PROXYL nitroxide radicals, dynamic fluorescence quenching

## Abstract

The influence of the stable 2,2,6,6-tetramethylpiperidinyl-*N*-oxyl (TEMPO) nitroxide and its six C4-substituted derivatives, as well as two C3-substituted analogues of 2,2,5,5-tetramethylpyrrolidynyl-*N*-oxyl (PROXYL) nitroxide on the chosen fluoroquinolone antibiotics (marbofloxacin, ciprofloxacin, danofloxacin, norfloxacin, enrofloxacin, levofloxacin and ofloxacin), has been examined in aqueous solutions by UV absorption as well as steady-state and time-resolved fluorescence spectroscopies. The mechanism of fluorescence quenching has been specified and proved to be purely dynamic (collisional) for all the studied systems, which was additionally confirmed by temperature dependence experiments. Moreover, the selected quenching parameters—that is, Stern–Volmer quenching constants and bimolecular quenching rate constants—have been determined and explained. The possibility of electron transfer was ruled out, and the quenching was found to be diffusion-limited, being a result of the increase in non-radiative processes. Furthermore, as the chosen nitroxides affected the fluorescence of fluoroquinolone antibiotics in different ways, an influence of the structure and the type of substituents in the molecules of both fluoroquinolones and stable radicals on the quenching efficiency has been determined and discussed. Finally, the impact of the solvent’s polarity on the values of bimolecular quenching rate constants has been explained. The significance of the project comes from many applications of nitroxides in chemistry, biology and industry.

## 1. Introduction

The compounds 2,2,6,6-tetramethylpiperidinyl-*N*-oxyl (TEMPO), 2,2,5,5-tetramethylpyrrolidynyl-*N*-oxyl (PROXYL) and their derivatives—because of a large resonance and steric effects—are very stable [1]. These membrane-permeable nitroxides exhibit efficient protection of cells and tissues from damages related to an overproduction of free radicals (among others nitrogen dioxide [2]), which is due to the oxidative [3] and nitrative stress conditions [4]. This stems from the fact that they are able to catalyze the superoxide dismutation (mimic activity) [5], as well as detoxify carbon-centered [6], hydroxyl [7] and peroxyl radicals [8]. Furthermore, profluorescent nitroxides have been previously reported as probes for the detection of free radicals, as well as damage mediated by these species, for example acute pancreatitis and chronic splanchnic ischemia [9]. A variety of TEMPO and PROXYL analogues is also used as—among others—spin labels [10,11], radical scavengers [12], lipid oxidants [13], polymerization inhibitors [14], candidates for development as antiarrhythmic preventive drugs [15,16] or catalysts for chemical oxidation of alcohols [17]. The studies performed with the participation of these stable paramagnetic intermediates are known to result in some important phenomena, such as the quenching of photoexcited molecules [18] or formation of singlet oxygen [19]. Moreover, as the magnetic properties in the ground state are different before and after the photoexcitation, they seem to be attractive for controlling magnetic properties by light [20].

The fluoroquinolone antibiotics are a class of fully synthetic, broad-spectrum antibacterial agents with a unique mechanism of action and wide clinical use, structurally related to nalidixic acid and oxolinic acid [21]. Their activities result from the inhibition of various kinds of bacterial enzymes (among others DNA gyrase and topoisomerase IV) involved in the control of DNA synthesis and are vital for chromosome function, replication and transcription [22,23]. Fluoroquinolones are generally highly effective against both aerobic Gram (−) and many Gram (+) bacteria [24] and, thus, very helpful in the treatment of a wide range of infections, including urinary, bone-joint, enteral, soft tissue and respiratory tract infections, typhoid fever, prostatitis, blood poisoning, sinusitis, gonorrhea and bacterial gastroenteritis [25]. On the other hand, there is great concern for the fluoroquinolones that are widely used and at the same time are not readily biodegradable by microorganisms [26]. Different generations of fluoroquinolone antibiotics have been already developed to enlarge the spectrum of their activity and to solve problems of their resistance [27]. Since it has been proven that various types of fluoroquinolones—depending on substituents in the structure—exhibit quite different antibacterial responses and stability, it became very important to study the physical and chemical properties of these molecules in various environments [28]. Although the mechanism of action of all fluoroquinolones is very similar, there are many significant differences in their antimicrobial spectrum of activity, pharmacokinetic characteristics, safety profiles, transport characteristics and complexing properties—mainly because of a diversity of functional groups in antibiotics molecules [29]. All these prove that a precise understanding of the interaction mechanisms between antibiotics and other compounds is essential and of great importance.

In the following study, spectroscopic techniques, namely UV absorption and steady-state fluorescence spectroscopy, supported by time-resolved fluorescence lifetimes measurements, were used to investigate the interactions between different fluoroquinolones and selected TEMPO and PROXYL derivatives in aqueous solutions. Since all nitroxides were found to decrease the fluorescence intensity of the chosen antibiotics, while individual fluoroquinolones are known to exhibit particular properties in relation to their chemical structures [30], the main goal of the studies was to understand the mechanism of quenching involved in these systems as well as to determine whether the structure of both the fluorophore and the quencher has an influence on the observed fluorescence quenching efficiency.

## 2. Results and Discussion

For all the studied fluoroquinolone antibiotics, their UV absorption and fluorescence emission spectra were recorded under the action of the chosen nine nitroxide radicals. As similar observations were made for all 63 combinations, in the following paper, the spectra are presented, and their changes are explained on the example of danofloxacin and 4-hydroxy-TEMPO—the system in case of which the most effective fluorescence quenching was observed. Figure S1A in Supplementary Materials shows UV absorption spectra of the aqueous solution of danofloxacin (10 µM) in the presence of increasing amounts of 4-hydroxy-TEMPO, while Figure S1B presents the spectra of pure 4-hydroxy-TEMPO at analogous concentrations in aqueous solutions. From the inspection of these spectra, it can be clearly observed that in the studied range of concentrations, 4-hydroxy-TEMPO exhibits no influence on the UV spectrum of danofloxacin. When the concentration of 4-hydroxy-TEMPO increases, the band with a maximum at 243 nm (attributed to the presence of the nitroxide) gradually appears with no impact on the bands attributed to the antibiotic (namely 282 and 344 nm). Figure S2 in Supplementary Materials shows the fluorescence emission spectra of the excited at 340 nm pure danofloxacin and one spectrum in the presence of increasing concentrations of 4-hydroxy-TEMPO. The addition of the nitroxide immediately affects solely the fluorescence intensity of the fluoroquinolone with no changes in the shape of its spectrum and the shift of the band with maximum at 440 nm, which suggests no change in hydrophobicity of the microenvironment of the potential drug binding region [31]. All these findings (from both UV absorption and fluorescence emission measurements) prove that strictly physical interactions between danofloxacin and 4-hydroxy-TEMPO occur, with no option for a complex (also emissive exciplex) formation or photochemistry involved.

**Figure 1:**
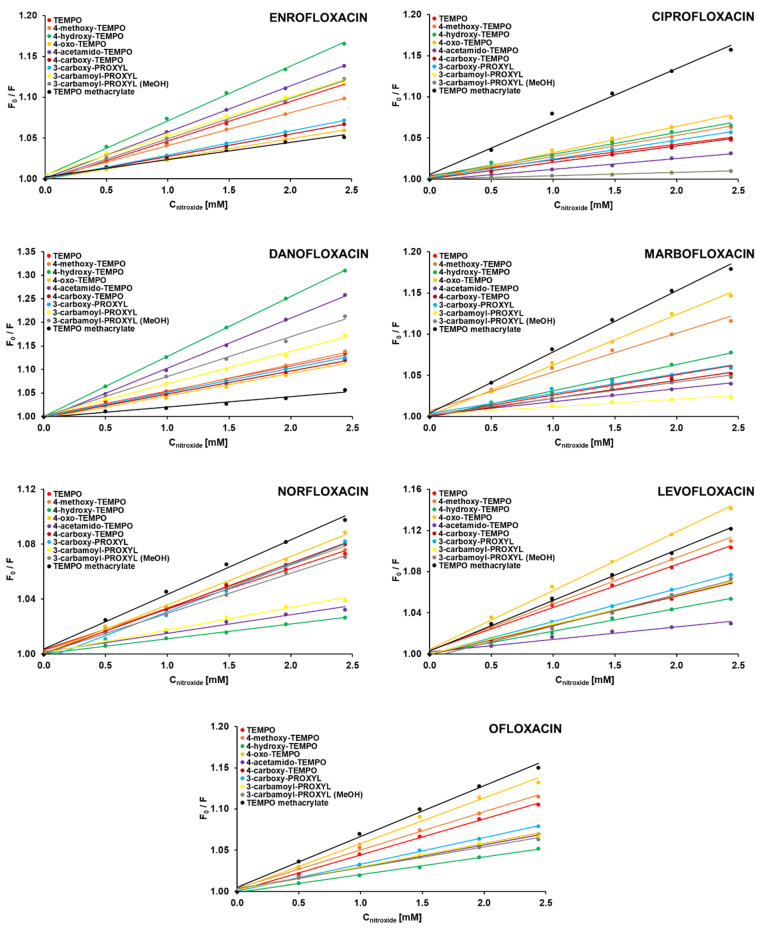
Stern–Volmer plots for the steady-state fluorescence quenching of the studied fluoroquinolones by different 2,2,6,6-tetramethylpiperidinyl-*N*-oxyl (TEMPO) and 2,2,5,5-tetramethylpyrrolidynyl-*N*-oxyl (PROXYL) derivatives in aqueous solutions at 20 °C. F_0_ and F are the fluorescence intensities of the fluoroquinolones in the absence and presence of the nitroxides, respectively. The results are shown as a mean of three independent experiments. In all experiments, standard deviations were less than 4%, and thus, for better clarity, error bars were omitted.

Figure 1 shows the Stern–Volmer plots obtained for the steady-state fluorescence quenching of the studied fluoroquinolones by various TEMPO and PROXYL derivatives in aqueous solutions. In the chosen range of nitroxides’ concentrations, there are no deviations from the observed linearity—the fluorescence quenching shows very clearly a linear dependence such as the one represented by the Stern–Volmer equation. Since it has been previously proven that 4-hydroxy-TEMPO impacts (shortens) on a fluorescence lifetime of selected fluoroquinolones [32], it indicates that dynamic, collisional fluorescence quenching occurred. Consequently, Table 1 presents the newly determined values of Stern–Volmer dynamic quenching constants (*K*_D_) along with linear correlation coefficients (R^2^) for Stern–Volmer plots and bimolecular quenching rate constants (*k*_q_) obtained for the studied systems. The latter ones were calculated based on determined values of fluorescence lifetimes, which were found to be in good agreement with literature data [33,34]. The postulated mechanism of fluorescence quenching corresponds well with the results obtained from UV absorption experiments, since collisions among the fluorophore and quencher molecules would not change the absorption spectrum. Additionally, the above finding has been confirmed by experiments at various temperatures. From the inspection of Figure S3 in Supplementary Materials, it can be observed that the increase in temperature results in greater values of Stern–Volmer constants (higher slopes). It is characteristic for dynamic quenching (the higher the temperature, the faster diffusion and, thus, more collisions)—contrary to static quenching, where the increase in the temperature favors dissociation of weakly bound complexes (higher temperature is likely to reduce the stability of the complexes, resulting in lower static quenching constants) [35]. The fact that stable nitroxides quench the fluorescence of the studied fluoroquinolones through the collisions is quite interesting, since a vast majority of scientific data reports the static quenching of these fluorophores by various quenchers [31,36,37,38,39,40].

To gain a deeper insight into the mechanism involved, it should be noted that there is no obvious correlation between the observed fluorescence quenching efficiency (expressed by the values of bimolecular quenching rate constants) and ionization energies of the studied quenchers [41] and antibiotics [32]. Thus, the mechanism of electron transfer can be excluded. Furthermore, it is hard to observe a relationship between *k*_q_ values and nitroxides’ molecular radii—probably because of comparable sizes of the chosen radicals and fluoroquinolones. Generally, it is more than likely that various factors simultaneously have an influence on the reported decrease in fluorescence. Being amphoteric compounds, fluoroquinolone antibiotics may occur in various forms related to the values of dissociation constants p*K*_a_—at pH near neutral they exist mainly as zwitterionic species [42]. Since at the same conditions nitroxide radicals may occur as neutral molecules as well as both negatively and positively charged ions, electrostatic interactions should not be ruled out. It has been confirmed by our previous studies, which revealed that better packing and access of a quencher to the electrically polarized molecule of a fluorophore increases the efficiency of fluorescence quenching [43]. Additionally, bimolecular quenching rate constants are of the same order of magnitude as diffusion rate constants obtained for studies performed in water [35]—thus, it is highly probable that the bimolecular process is diffusion-limited, caused by the increase in rate constants of non-radiative transitions, such as an internal conversion or an intersystem crossing. Interestingly, in the case of all studied fluoroquinolones, *k*_q_ values obtained for 3-carboxy-PROXYL are slightly higher than for 4-carboxy-TEMPO. Although the reasons for the observed difference between five-membered and six-membered analogues used as quenchers may come from the electron distribution, they are not clear, and evidently, there is a scope for further investigations. By looking at the results obtained for 3-carbamoyl-PROXYL in water and the same quencher in methanol, it can be concluded that the greater value of dielectric constant of the solvent (the more polar solvent), the lower efficiency of the fluorescence quenching of all fluoroquinolones. With a high probability, it is a consequence of two effects, one associated with preferential solvation and related changes in intermolecular interactions and the second one connected with the changes in environmental viscosity.

## 3. Materials and Methods

**Figure 2:**
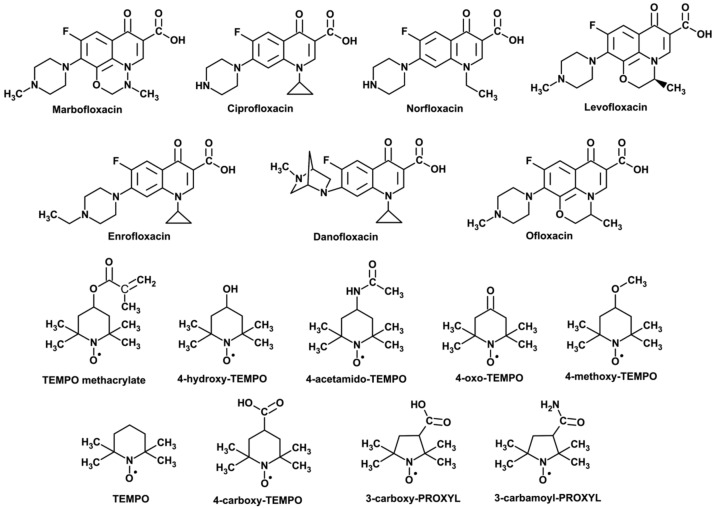
Molecular structures of the selected nitroxide radicals and fluoroquinolone antibiotics (with their commercial names).

All reagents, that is, the ones chosen for the studies of fluoroquinolone antibiotics (marbofloxacin, ciprofloxacin, danofloxacin, norfloxacin, enrofloxacin, levofloxacin and ofloxacin), TEMPO (2,2,6,6-tetramethylpiperidinyl-*N*-oxyl) and its C4-substituted derivatives (4-hydroxy-TEMPO, 4-methoxy-TEMPO, 4-carboxy-TEMPO, 4-acetamido-TEMPO, 4-oxo-TEMPO and TEMPO methacrylate) as well as two C3-substituted analogues of PROXYL (2,2,5,5-tetramethylpyrrolidynyl-*N*-oxyl), namely 3-carboxy-PROXYL and 3-carbamoyl-PROXYL, were purchased from Sigma Aldrich (Poland). All reagents were of purity no less than 97% and were used without further purification. The double-distilled water with a conductivity less than 0.18 μS∙cm^−1^ as well as methanol (purity >99.8%, water <0.1%) were used as solvents. The stock solutions of fluoroquinolones and nitroxides were prepared just before use by dissolving an appropriate amount of the substance in water (with the help of sonication if necessary; in one case, i.e., 3-carbamoyl-PROXYL, additionally in methanol). To avoid self-quenching or inner filter effects, the solutions of all antibiotics were prepared keeping the constant concentration (10 µM). In all fluorometric measurements, the optical density of the solutions did not exceed 0.1. The concentration of the stock solutions of TEMPO and PROXYL radicals was equal to 0.1 M. The molecular structures of the studied compounds are presented in Figure 2.

UV absorption spectra of the aqueous solutions of the studied fluoroquinolones (10 µM) were recorded on a Perkin Elmer Lambda 650 (Waltham, MA, USA) UV-Vis spectrophotometer (a slit—2 nm) at the temperature of 20 °C in the presence of all the selected TEMPO and PROXYL nitroxides used at concentrations 0–0.25 mM. 

Fluorescence emission spectra as well as all fluorescence intensity measurements were performed with the use of a Cary Eclipse Varian (Agilent, Santa Clara, CA, USA) spectrofluorometer—equipped with a temperature controller and a 1.0 cm multicell holder; excitation and emission slits −2.5 or 5 nm, depending on fluoroquinolone’s quantum yield—at the temperature of 20 °C (in one case, i.e., danofloxacin/4-hydroxy-TEMPO system, additionally at 30 and 40 °C) in the presence of all the selected TEMPO and PROXYL nitroxides used at concentrations 0–2.5 mM. Excitation wavelength in case of steady-state experiments for all fluoroquinolones was set at 340 nm, based on absorption spectra, which are presented in Figure S4 in Supplementary Materials. The fluorescence intensity values were always measured at the maximum of the emission (470 nm for marbofloxacin; 423 nm for ciprofloxacin; 428 nm for norfloxacin; 440 nm for danofloxacin; 437 nm for enrofloxacin; 468 nm for levofloxacin; 475 nm for ofloxacin; fluorescence emission spectra of all studied antibiotics are presented in Figure S5 in Supplementary Materials). In the performed titration experiments, 2 mL of each fluoroquinolone at 10 µM was titrated with five 10 μL aliquots of each nitroxide solution at 0.1 M, and after gentle stirring, the corresponding fluorescence intensity was measured. The Stern–Volmer equation was used to determine the type of fluorescence quenching involving the studied fluoroquinolones and nitroxides:

$$\frac{F_{0}}{F}=1+K_{SV}\left[Q\right]=1+\;k_{q}\tau_{0}\left[Q\right].$$

In this equation, *F*_0_ and *F* are the fluorescence intensities in the absence and presence of quencher, respectively; *k*_q_ is the bimolecular quenching rate constant; τ_0_ is the lifetime of the fluorophore in the absence of quencher; [*Q*] is the concentration of quencher. The Stern–Volmer quenching constant is given by *K*_SV_ = *k*_q_τ_0_. If the quenching is known to be dynamic, the Stern–Volmer constant is represented by *K*_D_. In that case, a linear plot of *F*_0_/*F* versus [*Q*] yields an intercept of one on the *y*-axis and a slope equal to *K*_D_. Furthermore, for the system, in the case of which the most significant fluorescence intensity changes were observed (danofloxacin + 4-hydroxy-TEMPO)—to confirm the observed fluorescence quenching mechanism—the decrease in fluorescence intensity of the antibiotic under the action of the nitroxide was recorded at three different temperatures, namely 20, 30 and 40 °C with an accuracy of ±0.01 °C. Since the fluorescence measurements may be adversely affected by the inner-filter effects [44,45]—which can be caused by the absorption by the nitroxides of light at the excitation and emission wavelengths of fluoroquinolones—all experimentally determined values of fluorescence intensities were corrected using the following equation:

$$F_{\mathrm{corr}}=F_{\mathrm{obs}}\cdot 10^{\frac{A_{\mathrm{ex}}+A_{\mathrm{em}}}{2}},$$

where F_corr_ and F_obs_ are the corrected and observed fluorescence intensities, respectively; whereas, A_ex_ and A_em_ are the sum of the absorbance of the appropriate antibiotic and the nitroxide radical at the excitation and emission wavelength, respectively.

Fluorescence lifetimes of all studied fluoroquinolone antibiotics were measured using the single photon counting technique (at the maximum of emission chosen by monochromator; c = 10 µM) with the use of a FluoTime 300 (PicoQuant, Berlin, Germany) high performance fluorescence lifetime spectrometer (PicoQuant) at 20 °C. The excitation source was subnanosecond pulsed diode: PLS-340 nm.

## 4. Conclusions

The paper presents the results of our studies on the interactions between chosen fluoroquinolone antibiotics and various TEMPO and PROXYL nitroxides. It has been proven that fluorescence of all investigated fluoroquinolones is quenched by the stable radicals. The results of steady-state fluorescence and time-resolved measurements made it possible to determine bimolecular quenching rate constants for all the systems studied. The mechanism of fluorescence quenching was found to be totally dynamic/collisional and diffusion-limited due to the increase in non-radiative processes, such as the internal conversion or intersystem crossing. Furthermore, as the chosen nitroxides affected fluorescence of fluoroquinolone antibiotics in different ways, an influence of the structure and the type of substituents in the molecules of both fluoroquinolones and stable radicals on the quenching efficiency has been determined and discussed. The performance of fluorescence quenching experiments in pure water and methanol enabled us to state that the more polar solvent, the lower efficiency of quenching, which may arise from the solvation and/or viscosity effect.

## Figures and Tables

**Table 1:** Stern–Volmer dynamic quenching constants (*K*_D_), linear correlation coefficients (R^2^) and bimolecular quenching rate constants (*k*_q_) recovered for the steady-state fluorescence quenching of the studied fluoroquinolones by various TEMPO and PROXYL nitroxides in aqueous solutions at 20 °C. The results are shown as a mean ±standard deviation (S.D.) of three independent experiments.

Nitroxide Radical	Fluoroquinolone (Fluorescence Lifetime ± S.D.)	*K*_D_ ± S.D. [mol^−1^·dm^3^]	R^2^	*k*_q_ ± S.D. [mol^−1^·dm^3^·s^−1^]
TEMPO	Enrofloxacin (1.95 ± 0.02 ns)	48.32 ± 1.01	0.998	(2.48 ± 0.08) × 10^10^
4-methoxy-TEMPO		40.29 ± 0.24	1.000	(2.07 ± 0.03) × 10^10^
4-hydroxy-TEMPO		66.92 ± 1.62	0.998	(3.43 ± 0.12) × 10^10^
4-oxo-TEMPO		24.71 ± 0.54	0.998	(1.27 ± 0.04) × 10^10^
4-acetamido-TEMPO		56.48 ± 0.34	1.000	(2.90 ± 0.05) × 10^10^
4-carboxy-TEMPO		27.33 ± 0.24	1.000	(1.40 ± 0.03) × 10^10^
3-carboxy-PROXYL		29.52 ± 0.14	1.000	(1.51 ± 0.02) × 10^10^
3-carbamoyl-PROXYL		47.75 ± 1.93	0.994	(2.45 ± 0.12) × 10^10^
3-carbamoyl-PROXYL (MeOH)		49.26 ± 0.92	0.999	(2.53 ± 0.07) × 10^10^
TEMPO methacrylate		21.03 ± 1.18	0.988	(1.08 ± 0.07) × 10^10^
TEMPO	Ciprofloxacin (1.54 ± 0.01 ns)	18.93 ± 1.90	0.961	(1.23 ± 0.13) × 10^10^
4-methoxy-TEMPO		25.50 ± 1.02	0.994	(1.66 ± 0.08) × 10^10^
4-hydroxy-TEMPO		26.60 ± 1.70	0.984	(1.73 ± 0.12) × 10^10^
4-oxo-TEMPO		31.73 ± 1.45	0.992	(2.06 ± 0.11) × 10^10^
4-acetamido-TEMPO		13.07 ± 0.51	0.994	(8.49 ± 0.39) × 10^9^
4-carboxy-TEMPO		20.02 ± 0.88	0.992	(1.30 ± 0.07) × 10^10^
3-carboxy-PROXYL		23.10 ± 0.62	0.997	(1.50 ± 0.05) × 10^10^
3-carbamoyl-PROXYL		4.11 ± 0.13	0.996	(2.67 ± 0.10) × 10^9^
3-carbamoyl-PROXYL (MeOH)		4.15 ± 0.18	0.993	(2.69 ± 0.13) × 10^9^
TEMPO methacrylate		64.30 ± 3.35	0.989	(4.18 ± 0.24) × 10^10^
TEMPO	Danofloxacin (5.97 ± 0.05 ns)	52.94 ± 1.67	0.996	(8.87 ± 0.35) × 10^9^
4-methoxy-TEMPO		57.51 ± 1.81	0.996	(9.63 ± 0.38) × 10^9^
4-hydroxy-TEMPO		127.3 ± 0.3	1.000	(2.13 ± 0.02) × 10^10^
4-oxo-TEMPO		47.77 ± 2.35	0.990	(8.00 ± 0.46) × 10^9^
4-acetamido-TEMPO		106.6 ± 1.5	0.999	(1.79 ± 0.04) × 10^10^
4-carboxy-TEMPO		48.96 ± 0.66	0.999	(8.20 ± 0.18) × 10^9^
3-carboxy-PROXYL		51.46 ± 0.47	1.000	(8.62 ± 0.15) × 10^9^
3-carbamoyl-PROXYL		68.56 ± 1.89	0.997	(1.15 ± 0.04) × 10^10^
3-carbamoyl-PROXYL (MeOH)		84.70 ± 2.50	0.996	(1.42 ± 0.05) × 10^10^
TEMPO methacrylate		22.00 ± 1.86	0.972	(3.69 ± 0.34) × 10^9^
TEMPO	Marbofloxacin (3.54 ± 0.02 ns)	24.58 ± 0.65	0.997	(6.94 ± 0.22) × 10^9^
4-methoxy-TEMPO		47.07 ± 2.84	0.986	(1.33 ± 0.09) × 10^10^
4-hydroxy-TEMPO		31.86 ± 0.82	0.997	(9.00 ± 0.28) × 10^9^
4-oxo-TEMPO		61.13 ± 1.58	0.997	(1.73 ± 0.05) × 10^10^
4-acetamido-TEMPO		15.86 ± 0.83	0.989	(4.48 ± 0.26) × 10^9^
4-carboxy-TEMPO		21.92 ± 0.68	0.996	(6.19 ± 0.23) × 10^9^
3-carboxy-PROXYL		24.05 ± 2.09	0.971	(6.79 ± 0.63) × 10^9^
3-carbamoyl-PROXYL		9.13 ± 1.07	0.948	(2.58 ± 0.32) × 10^9^
3-carbamoyl-PROXYL (MeOH)		19.85 ± 1.14	0.987	(5.61 ± 0.35) × 10^9^
TEMPO methacrylate		74.23 ± 2.31	0.996	(2.10 ± 0.08) × 10^10^
TEMPO	Norfloxacin(1.62 ± 0.02 ns)	29.56 ± 1.22	0.993	(1.82 ± 0.10) × 10^10^
4-methoxy-TEMPO		31.24 ± 1.12	0.995	(1.93 ± 0.09) × 10^10^
4-hydroxy-TEMPO		10.81 ± 0.21	0.998	(6.67 ± 0.21) × 10^9^
4-oxo-TEMPO		35.47 ± 0.97	0.997	(2.19 ± 0.09) × 10^10^
4-acetamido-TEMPO		13.57 ± 0.87	0.984	(8.38 ± 0.64) × 10^9^
4-carboxy-TEMPO		33.06 ± 0.39	0.999	(2.04 ± 0.05) × 10^10^
3-carboxy-PROXYL		34.46 ± 1.33	0.994	(2.13 ± 0.11) × 10^10^
3-carbamoyl-PROXYL		16.54 ± 0.74	0.992	(1.02 ± 0.06) × 10^10^
3-carbamoyl-PROXYL (MeOH)		29.20 ± 0.54	0.999	(1.80 ± 0.06) × 10^10^
TEMPO methacrylate		39.74 ± 1.52	0.994	(2.45 ± 0.12) × 10^10^
TEMPO	Levofloxacin(7.10 ± 0.06 ns)	41.65 ± 1.34	0.996	(5.87 ± 0.24) × 10^9^
4-methoxy-TEMPO		45.11 ± 1.65	0.995	(6.35 ± 0.29) × 10^9^
4-hydroxy-TEMPO		22.12 ± 0.67	0.996	(3.12 ± 0.12) × 10^9^
4-oxo-TEMPO		57.08 ± 1.78	0.996	(8.04 ± 0.32) × 10^9^
4-acetamido-TEMPO		12.24 ± 1.03	0.978	(1.72 ± 0.16) × 10^9^
4-carboxy-TEMPO		28.40 ± 0.50	0.999	(4.00 ± 0.10) × 10^9^
3-carboxy-PROXYL		31.78 ± 0.18	1.000	(4.48 ± 0.06) × 10^9^
3-carbamoyl-PROXYL		28.62 ± 0.64	0.998	(4.03 ± 0.12) × 10^9^
3-carbamoyl-PROXYL (MeOH)		30.64 ± 1.30	0.993	(4.32 ± 0.22) × 10^9^
TEMPO methacrylate		49.06 ± 1.15	0.998	(6.91 ± 0.22) × 10^9^
TEMPO	Ofloxacin(7.01 ± 0.05 ns)	43.74 ± 0.81	0.999	(6.24 ± 0.16) × 10^9^
4-methoxy-TEMPO		46.56 ± 1.45	0.996	(6.64 ± 0.25) × 10^9^
4-hydroxy-TEMPO		21.33 ± 0.51	0.998	(3.04 ± 0.09) × 10^9^
4-oxo-TEMPO		55.53 ± 2.27	0.993	(7.92 ± 0.38) × 10^9^
4-acetamido-TEMPO		27.10 ± 0.81	0.996	(3.87 ± 0.14) × 10^9^
4-carboxy-TEMPO		28.11 ± 0.68	0.998	(4.00 ± 0.13) × 10^9^
3-carboxy-PROXYL		32.45 ± 0.36	1.000	(4.63 ± 0.08) × 10^9^
3-carbamoyl-PROXYL		25.35 ± 1.27	0.990	(3.62 ± 0.21) × 10^9^
3-carbamoyl-PROXYL (MeOH)		27.90 ± 1.07	0.994	(3.98 ± 0.18) × 10^9^
TEMPO methacrylate		61.78 ± 2.26	0.995	(8.81 ± 0.39) × 10^9^

## Data Availability

The data presented in this study are available on reasonable request from the corresponding author.

## Supplementary Materials

The following are available online at https://www.mdpi.com/1422-0067/22/2/885/s1. Figure S1: UV absorption spectra of danofloxacin (10 µM) in the presence of increasing concentrations of 4-hydroxy-TEMPO (0–0.25 mM in the measured sample) (**A**) and pure 4-hydroxy-TEMPO at analogous concentrations in aqueous solution (**B**), Figure S2: Fluorescence emission spectra of danofloxacin (10 µM) in the presence of increasing concentrations of 4-hydroxy-TEMPO (0–2.5 mM in the measured sample); λ_ex_ = 340 nm, Figure S3: Stern–Volmer plots from the steady-state fluorescence quenching of danofloxacin (10 µM) by 4-hydroxy-TEMPO in aqueous solutions at different temperatures (20, 30 and 40 °C); λ_ex_ = 340 nm. The results are shown as mean ± standard deviation (S.D.) of three independent experiments. In all experiments, standard deviations were less than 4%, and thus, for a better clarity, error bars were omitted, Figure S4: UV absorption spectra of the studied fluoroquinolone antibiotics (10 µM), Figure S5: Fluorescence emission spectra of the studied fluoroquinolone antibiotics (10 µM); λ_ex_ = 340 nm.
