# Supplementary material for: Fluorescence Quenching Studies on the Interactions between Chosen Fluoroquinolones and Selected Stable TEMPO and PROXYL Nitroxides
Source: Int J Mol Sci. 2021 Jan 17;22(2):885. doi: 10.3390/ijms22020885 (PMC7830230; doi:10.3390/ijms22020885)
Supplement: Supplementary file 1 [file ijms-22-00885-s001.pdf]

Supplementary Materials

Fluorescence quenching studies on the interactions between chosen fluoroquinolones and selected stable TEMPO and PROXYL nitroxides

Krzysztof Żamojć*, Irena Bylińska, Wiesław Wiczk, Lech Chmurzyński

Faculty of Chemistry, University of Gdańsk, Wita Stwosza 63, 80-308 Gdańsk, Poland

*Corresponding author:
E-mail address: krzysztof.zamojc@ug.edu.pl,
telephone number: (+48 58) 523 50 57,
fax number: (+48 58) 523 50 12

Contents

Figure S1. UV absorption spectra of danofloxacin (10 μM) in the presence of increasing concentrations of 4-hydroxy-TEMPO (0 – 0.25 mM in the measured sample) (A) and pure 4-hydroxy-TEMPO at analogous concentrations in aqueous solution (B).

Figure S2. Fluorescence emission spectra of danofloxacin (10 μM) in the presence of increasing concentrations of 4-hydroxy-TEMPO (0 – 2.5 mM in the measured sample); λ_{ex} = 340 nm.

Figure S3. Stern-Volmer plots from the steady-state fluorescence quenching of danofloxacin (10 μM) by 4-hydroxy-TEMPO in aqueous solutions at different temperatures (20, 30, and 40°C); λ_{ex} = 340 nm. The results are shown as a mean ± standard deviation (S.D.) of three independent experiments. In all experiments standard deviations were less than 4% and thus, for a better clarity, error bars were omitted.

Figure S4. UV absorption spectra of the studied fluoroquinolone antibiotics (10 μM).

Figure S5. Fluorescence emission spectra of the studied fluoroquinolone antibiotics (10 μM); λ_{ex} = 340 nm.

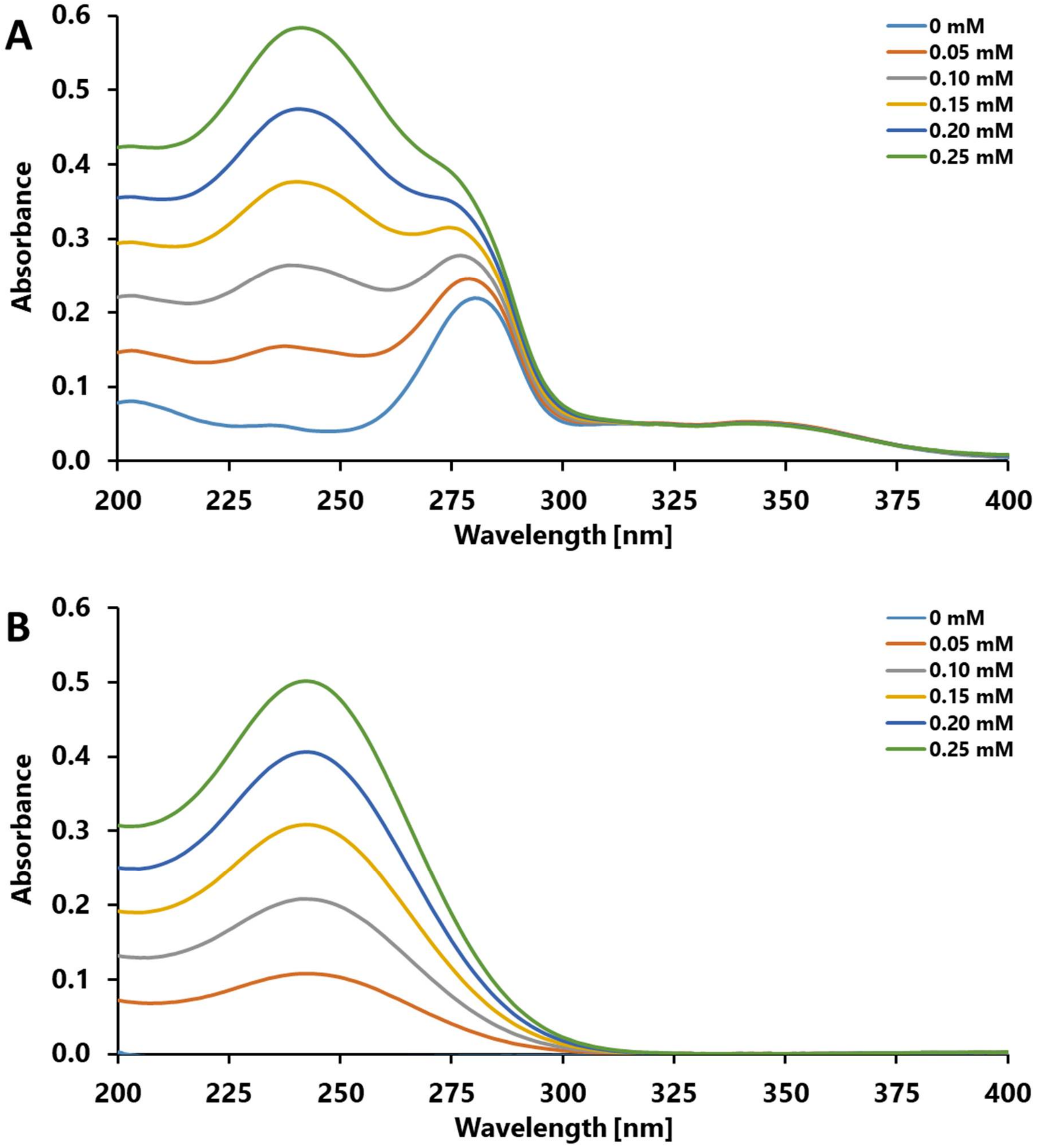

Figure S1. UV absorption spectra of danofloxacin (10 μM) in the presence of increasing concentrations of 4-hydroxy-TEMPO (0 – 0.25 mM in the measured sample) (A) and pure 4-hydroxy-TEMPO at analogous concentrations in aqueous solution (B).

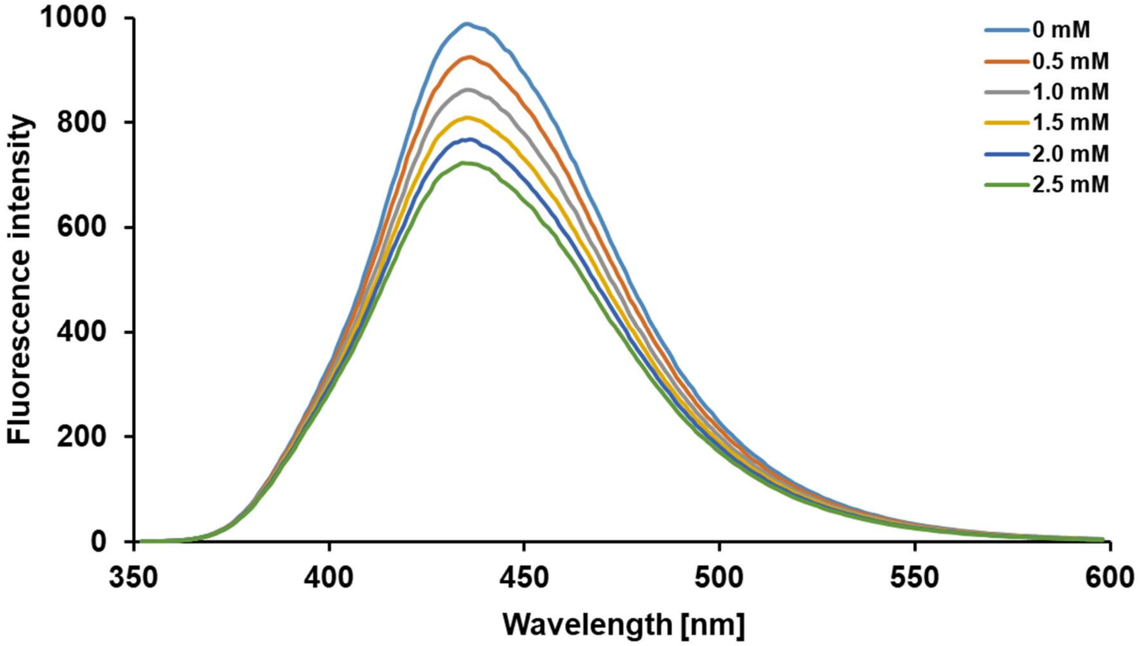

Figure S2. Fluorescence emission spectra of danofloxacin (10 μM) in the presence of increasing concentrations of 4-hydroxy-TEMPO (0 – 2.5 mM in the measured sample); λ_{ex} = 340 nm.

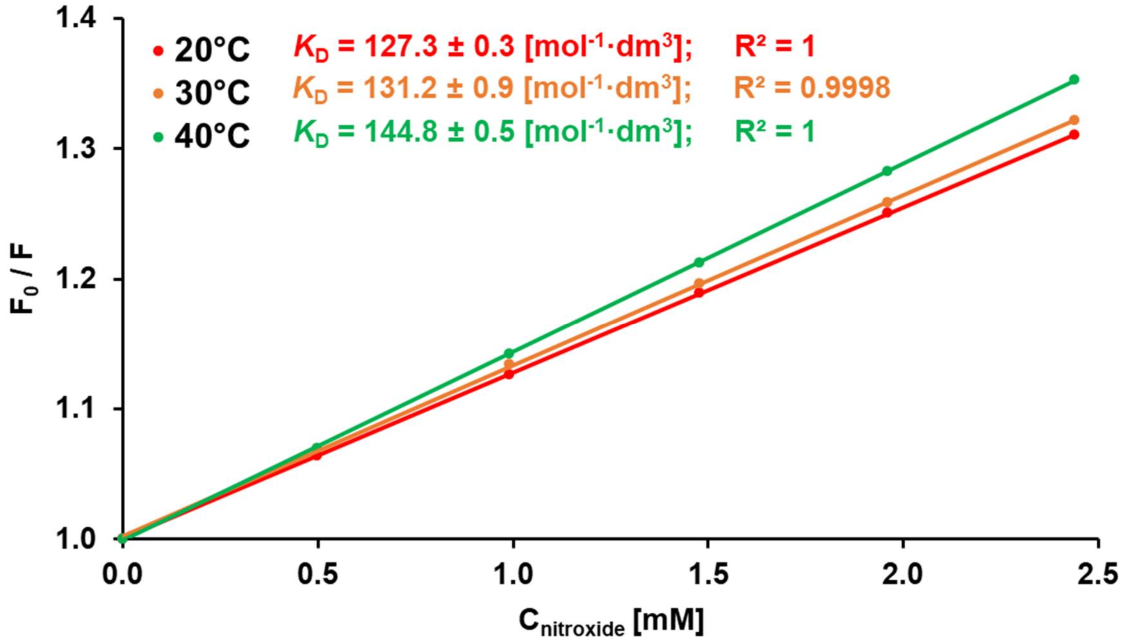

Figure S3. Stern-Volmer plots from the steady-state fluorescence quenching of danofloxacin (10 μM) by 4-hydroxy-TEMPO in aqueous solutions at different temperatures (20, 30, and 40°C); λ_{ex} = 340 nm. The results are shown as a mean ± standard deviation (S.D.) of three independent experiments. In all experiments standard deviations were less than 4% and thus, for a better clarity, error bars were omitted.

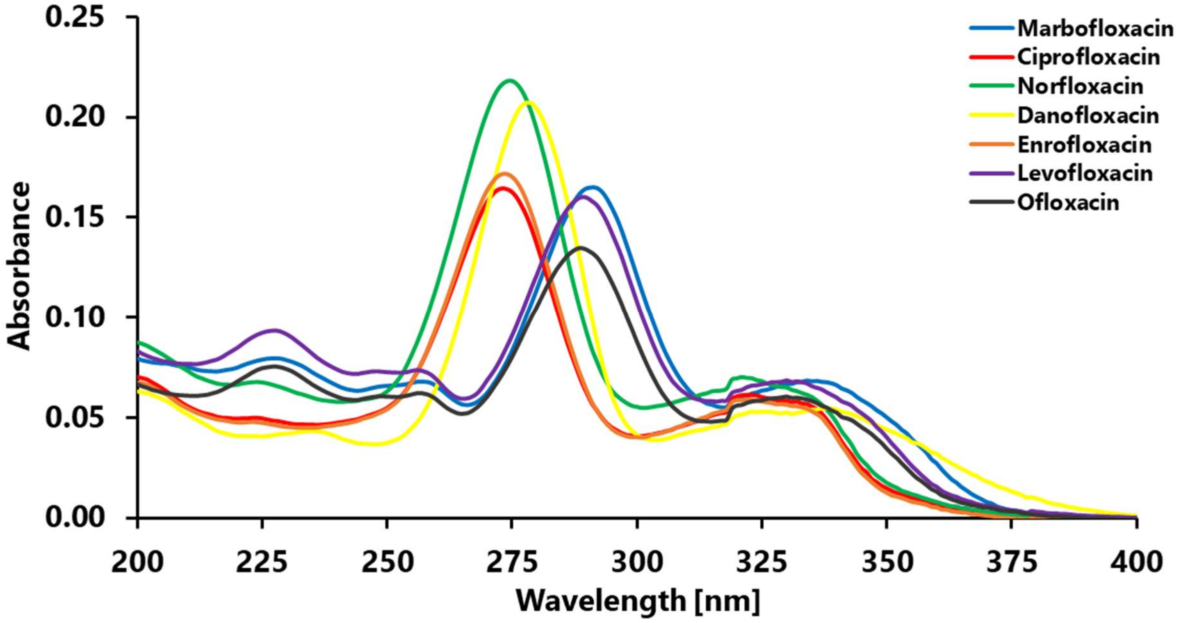

Figure S4. UV absorption spectra of the studied fluoroquinolone antibiotics (10 μM).

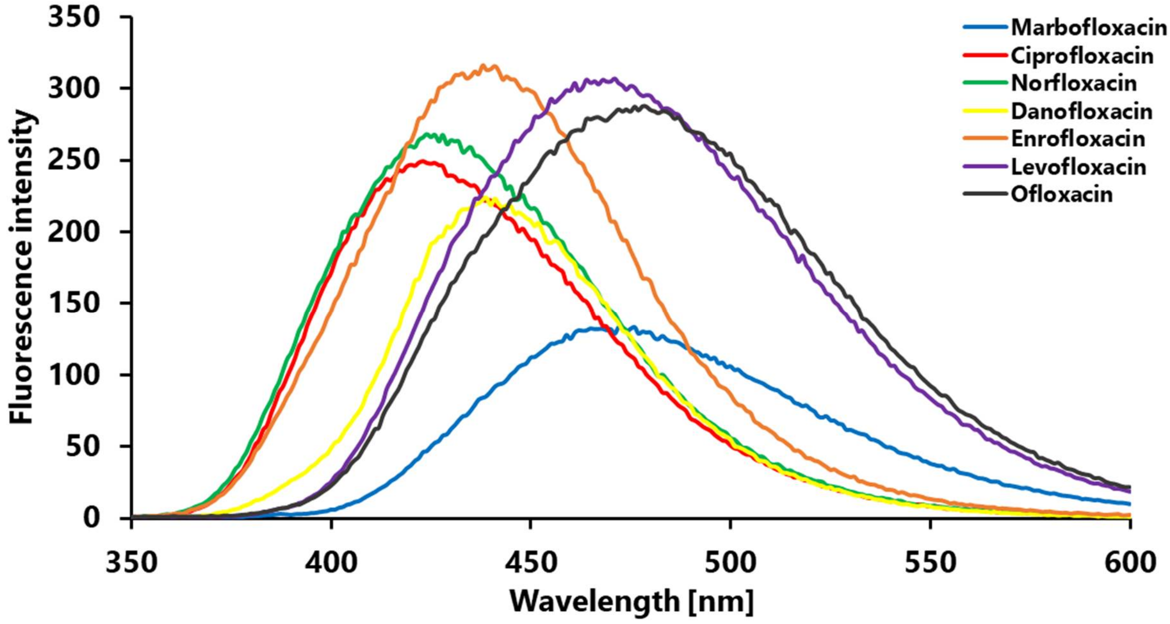

Figure S5. Fluorescence emission spectra of the studied fluoroquinolone antibiotics (10 μM); λ_{ex} = 340 nm.
